# Radiation therapy for cervical cancer in Uganda: a practice guideline

**DOI:** 10.3332/ecancer.2025.1999

**Published:** 2025-09-25

**Authors:** Solomon Kibudde, Awusi Kavuma, Bonny Abal, Moses Fredrick Katumba, Cissy Bangidde Namutale, Daniel Kanyike, Israel Luutu

**Affiliations:** 1 Division of Radiation Oncology, Uganda Cancer Institute, PO Box 3935, Kampala, Uganda; 2 Department of Medicine, College of Health Sciences, Makerere University, PO Box 7072, Kampala, Uganda

**Keywords:** radiotherapy, cervical cancer, guidelines, Uganda

## Abstract

**Introduction:**

Radiation therapy (RT) is crucial in the management of cervical cancer, particularly in resource-limited settings where most patients present with advanced-stage disease. Advances in external beam radiotherapy (EBRT) planning and delivery techniques, brachytherapy (BT) and systemic therapy necessitate context-adapted guidelines to standardise care. We developed a clinical practice guideline to improve and to harmonise the multidisciplinary management of cervical cancer in Uganda.

**Methods:**

A multidisciplinary team of Radiation Oncologists, Medical Physicists and Radiation therapists developed the guideline using a modified Delphi process. The guideline was externally reviewed by experts from the International Gynaecological Radiation Oncology Consortium.

**Results:**

All newly diagnosed patients should undergo multisciplinary evaluation prior to radiotherapy. For early-stage cervical cancer, adjuvant radiotherapy after hysterectomy is indicated in women with intermediate-risk factors, while concurrent cisplatin-based chemoradiation is indicated in women with high-risk factors. The standard EBRT dose is 45–50 Gy; women with vaginal and/or parametrial disease should receive adjuvant vaginal vault BT to achieve an equivalent dose in 2 Gy (EQD2) of 60 Gy. For locally advanced cervical cancer, the standard of care is pelvic EBRT (45–50 Gy in 25 fractions) with concurrent cisplatin (40 mg/m^2^ weekly for 5–6 cycles) followed by image-guided adaptive brachytherapy delivering 24–28 Gy in 3–4 fractions to achieve an EQD2 of 80–85 Gy for small tumours and 85–90 Gy for large tumours. The overall treatment time should not exceed 56 days. In recurrent disease, management depends on the location of the recurrence and the interval since the previous RT. In metastatic disease, palliative RT is directed to symptomatic sites.

**Conclusion:**

This clinical practice guideline offers evidence-informed, context-specific recommendations for the use of EBRT and BT in cervical cancer management in Uganda. It aims to harmonise the role of RT within multidisciplinary care pathways.

## Introduction

Cervical cancer ranks as the fourth most prevalent cancer in women, contributing significantly to both incidence and mortality rates. In 2022, it was estimated that there were approximately 660,000 new cases and 350,000 deaths globally [1]. The burden of cervical cancer is highest in low-and middle-income countries (LMICs). In 2020, the highest age-standardised incidence rates for cervical cancer were observed in Eastern Africa, with 40 cases per 100,000 women [2]. In Uganda, cervical cancer is the leading cause of cancer-related incidence and mortality, with an estimated 6,900 new cases and 4,800 deaths reported in 2022 [3]. Squamous cell carcinomas account for approximately 80% of all cervical cancers, and most patients present with locally advanced stages at diagnosis, leading to poor treatment outcomes [4, 5].

Radiation therapy (RT) is crucial for the effective management of cervical cancer. Over the past two decades, there have been significant advancements in external beam radiation therapy (EBRT) treatment planning and delivery techniques, brachytherapy (BT), systemic therapy and automation with artificial intelligence (AI) [6–10]. However, access to advanced radiotherapy technologies remains challenging for most patients in resource-limited settings, where the burden of cervical cancer is highest. Several strategies to address access to radiotherapy for cervical cancer have been proposed, including the use of hypofractionated EBRT [11, 12] and the emerging role of induction chemotherapy [13].

To standardise the use of RT for cervical cancer in Uganda, we have developed a clinical practice guideline to improve and to harmonise the management of patients with cervical cancer within a multidisciplinary setting. This guideline provides an update to the current guideline published in 2005 [14], reflecting the transitions in radiotherapy techniques and technology while integrating recent evidence, in the era of modern radiotherapy practice [15]. Overall, the guideline is intended to enhance access to effective radiotherapy and promote evidence-based practices, while optimising the available resources.

## Methods

### Scope

This clinical practice guideline provides recommendations for the use of RT in the curative or palliative treatment of women diagnosed with all stages of invasive cervical cancer in Uganda. The recommendations are stratified into three broad categories, namely early cervical cancer, locally advanced cervical cancer (LACC), International Federation of Gynaecology and Obstetrics (FIGO stages IB3 – IVA) and metastatic and/or recurrent cervical cancer.

### Population

The target population comprises women aged 18 to 85 years with a histologically confirmed cervical squamous cell carcinoma or adenocarcinoma or adenosquamous carcinoma and FIGO stages IA to IVB, irrespective of human immunodeficiency virus (HIV) co-infection. Women with rare histological diagnoses such as small cell carcinoma, neuroendocrine tumour, lymphoma, sarcoma or melanoma, non-invasive and benign diseases of the cervix are excluded from the scope of this guideline.

### Guideline development

This guideline was compiled by radiation oncologists (ROs), medical physicists (MPs), radiation therapy technologists and radiation oncology nurses of the Division of Radiation Oncology at the Uganda Cancer Institute. The writing committee was led by an RO (Dr Solomon Kibudde) and met weekly for 2 months. Supplementary input was provided by experts from the International Gynaecological Radiation Oncology Consortium [16], led by a consultant clinical oncologist (Dr Alexandra Taylor), during weekly meetings from November 2024 to April 2025.

### Target users

The primary intended users of this guideline are RO, MPs, radiation therapists, dosimetrists, radiation oncology nurses, gynaecological oncologists, pathologists, palliative care practitioners and general medical and allied health practitioners involved in the diagnosis, treatment and follow-up of women with confirmed or suspected cervical cancer. Secondary intended users include researchers, policymakers, students and administrators.

### Literature search

A desk review was conducted on all search literature obtained using online electronic databases, including PubMed, Google Scholar, Scopus, Cochrane Library and Web of Science. The search was conducted in English, using MeSH terms generated from the guideline questions, such as 'Cervical Cancer' and ' RT' or 'Radiotherapy,' or ‘BT’ or ‘Treatment’ covering the period from 01 January 2000 to 30 April 2025. These data were reviewed and critically appraised by members of the writing committee.

### Evidence review

The literature was appraised based on the study design, strengths and limitations (such as sampling, blinding, allocation concealment and analysis methods), comparison groups, outcome variables and consistency of results across other studies.

### Formulation of recommendations

A modified Delphi technique [17] that follows a systematic and structured approach was employed to formulate the guideline recommendations. This process involved an extensive review of existing recommendations from other guidelines and adopting practices relevant to the Ugandan healthcare context. For each recommendation, we ensured alignment with the available supporting data regarding the benefits and harms to patients.

### External review

The guideline was reviewed by experts in Radiation Oncology and Medical Physics from the International Gynaecological Radiation Oncology Consortium [16], representing three large leading academic institutions. This review aimed to enhance the quality of the guideline and obtain feedback on the recommendations.

## Results

The guideline encompasses the use of EBRT and BT in the curative and palliative treatment of early-stage, locally-advanced and metastatic and/or recurrent cervical cancer in Uganda (Table 1).

### General recommendations

All patients should be evaluated by a multisciplinary team and must provide informed consent before commencement of any radiotherapy-related procedures. The clinical stage, according to the FIGO staging system (Table 2), performance status (Eastern Cooperative Oncology Group, ECOG) score, clinical-radiological drawing of the initial disease, HIV status and intent of treatment should be well documented. The staging evaluation will be complemented by a review of the patient's complete blood count, renal function tests, liver function tests, HIV serology results, CD4 T-cell count and/or HIV viral load in HIV-positive patients and a computed tomography (CT) scan of the chest, abdomen and pelvis. When available, results from a pelvic magnetic resonance imaging (MRI) and positron-emission tomography (PET)/CT scan will be reviewed. Cystoscopy and proctoscopy are not routinely recommended.

### Early-stage cervical cancer

**Figure 1.**
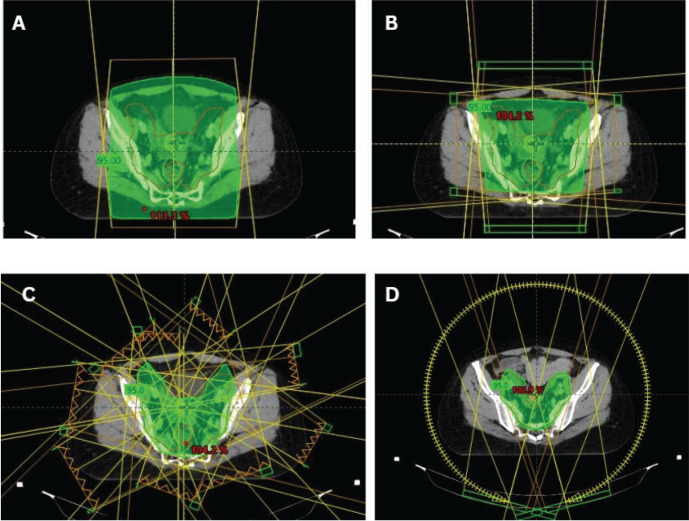
Cervical cancer EBRT techniques. (a): 2D radiotherapy beam arrangement with *AP-PA* opposing beams. (b): *3DCRT ‘*box*’* or 4-field beam arrangement. (c)*: 9-fields IMRT plan for cervical cancer*. (d): VMAT plan for cervical cancer.

For Stage IA1 disease without lymphovascular space invasion (LVSI), simple hysterectomy without lymphadenectomy is generally sufficient [18]. For stages IA2–IB2 and IIA1, hysterectomy with pelvic lymphadenectomy is recommended [19]. Patients with pathologically confirmed lymph node (LN) metastasis should be re-staged as FIGO IIIC1 per the 2018/2021 FIGO staging system. Post-operative radiotherapy alone is considered for patients with early cervical cancer (FIGO stages IA1–IB2 and IIA1) who have undergone hysterectomy and pathology findings reveals any of these intermediate-risk factors: LVSI plus deep one-third cervical stromal invasion and any tumour size or LVSI plus middle one-third cervical stromal invasion and tumour size ≥2 cm or LVSI plus superficial one-third cervical stromal invasion and tumour size <4 cm, noting that lesions ≥4 cm (IB3) represent locally advanced disease and should be managed accordingly or no LVSI plus middle or deep one-third cervical stromal invasion and tumour size ≥4 cm [20], referred to as the Sedlis criteria. Additionally, adenocarcinoma and adenosquamous histology have been considered an intermediate-risk factor [21]. The recommended EBRT dose is 45–50.4 Gy in 25–28 fractions (1.8 Gy per fraction), delivered 5 fractions per week over 5–5.5 weeks [20]. Advanced techniques such as image-guided radiotherapy utilising intensity-modulated radiotherapy (IMRT) and volumetric-modulated arc therapy (VMAT) are preferred over three-dimensional conformal radiotherapy (3DCRT) and two-dimensional radiotherapy (Figure 1). Patients with positive or close vaginal surgical margins should receive adjuvant vaginal vault BT dose of 10–15 Gy [22].

**Figure 2.**
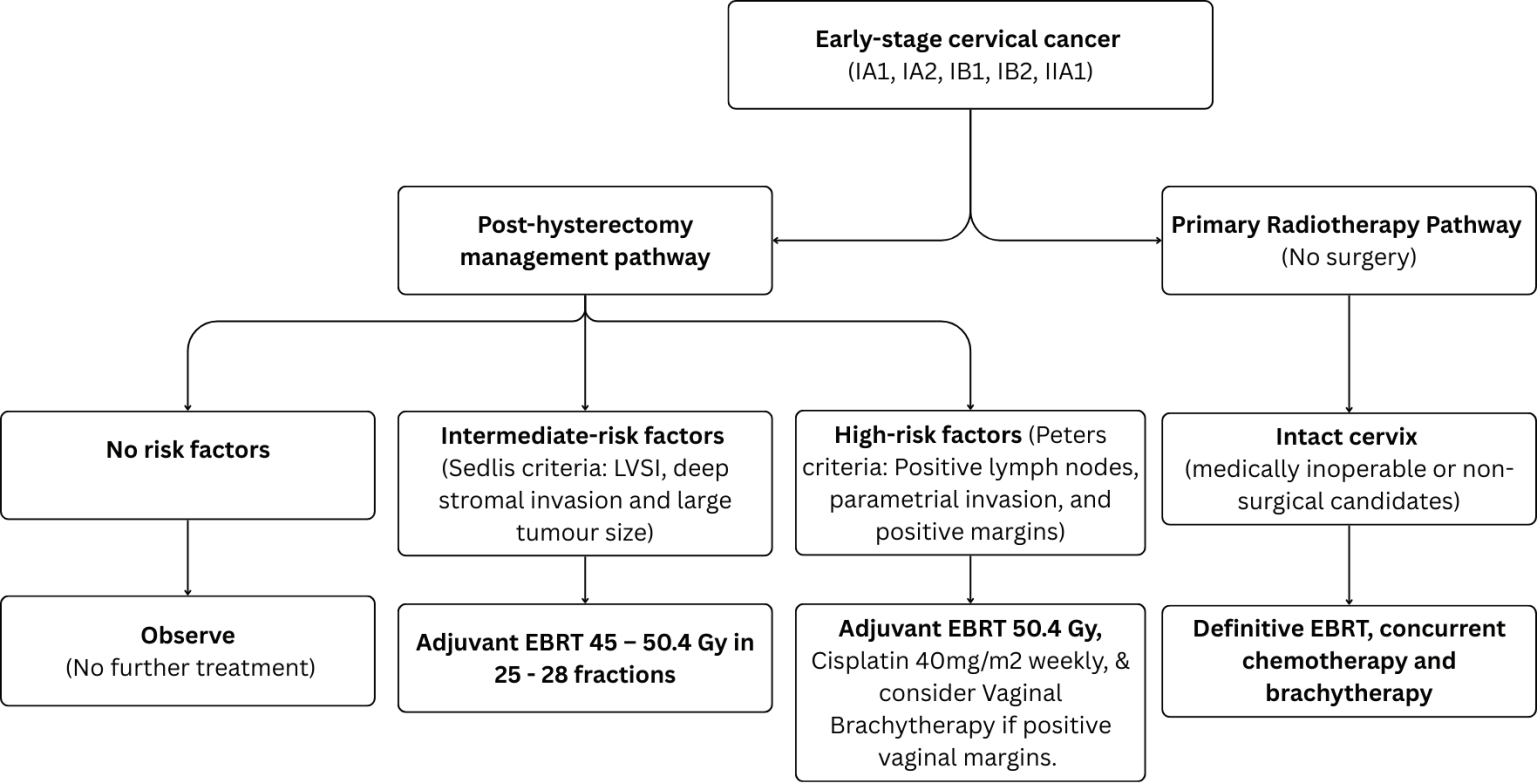
Use of RT in early-stage cervical cancer (IA1, IA2, IB1, IB2 and IIA1).

Adjuvant concurrent cisplatin-based chemoradiation improves overall survival and progression-free survival in women with high-risk factors referred to as Peter’s criteria, such as positive resection margins, LN involvement or parametrial invasion following radical hysterectomy [23]. The recommended EBRT dose is 50.4 Gy in 28 fractions (1.8 Gy per fraction), over 5.5 weeks [20] delivered using IMRT or VMAT using image-guidance with daily cone-beam CT or orthogonal kilovoltage images. Treatment with EBRT should commence within 6 to 8 weeks after surgery to maximise therapeutic efficacy and improve outcomes [24, 25]. The recommended BT dose scheme is 11 Gy in 2 fractions at 5.5 Gy per fraction to 5 mm below the vaginal surface or 18 Gy in 3 fractions at 6 Gy per fraction, to achieve an equivalent-dose in 2 Gy per fraction (EQD2) of 55 to 60 Gy and keeping the overall treatment time below 7 weeks [25] (Figure 2). However, for patients with central recurrence after hysterectomy, in the absence of interstitial BT, they should be offered a sequential EBRT boost to 66 Gy to the gross residual tumour by replanning to a smaller residual gross tumour after 4 weeks of the pelvic EBRT course.

### Locally advanced cervical cancer

**Figure 3.**
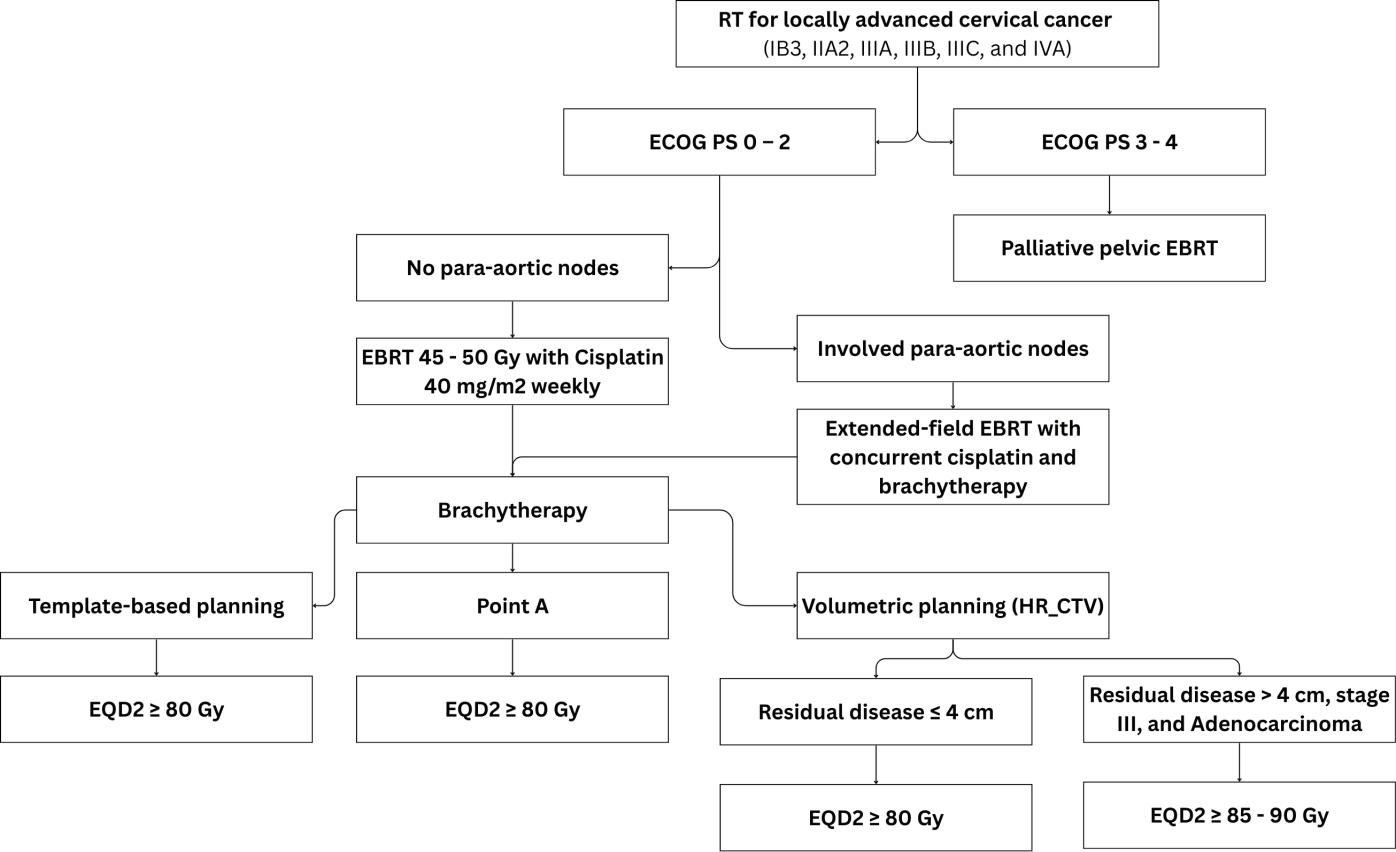
Use of RT in LACC (IB3, IIA2, IIIA, IIIB, IIIC and IVA).

The standard of care for LACC is definitive pelvic EBRT dose of 45–50 Gy in 25 fractions (1.8–2 Gy per fraction), delivered 5 fractions per week over 5 weeks with concurrent cisplatin (40 mg/m^2^) given weekly for 5–6 cycles, followed by Image-guided adaptive intracavitary/interstitial high-dose-rate (HDR) BT dose of 24–28 Gy in 3–4 fractions of 7–8 Gy, to achieve a total EBRT+BT dose of 80–85 Gy for small cervical tumours and 85–90 Gy for large cervical tumours or tumours with poor response to EBRT, adenocarcinoma and stage III at presentation [26]. The addition of concurrent chemotherapy results in a 6% improvement in 5-year survival compared with radiotherapy alone for women with LACC (HR 0·81, *p* < 0·001) [27]. While Cisplatin is the radiosensitiser of choice, carboplatin area under the curve 2 (AUC 2) weekly dose is an acceptable alternative to cisplatin among women with renal insufficiency (creatinine clearance ≤45 mL/min). Patients with pelvic and para-aortic LNs should receive EBRT boost to a dose of 55–60 Gy (EQD2) using simultaneous integrated boost or sequential boost technique, with consideration of contribution from BT (Figure 3).

Also, patients with involved para-aortic, common iliac LN and ≥3 involved pelvic LNs have a significant risk for para-aortic nodal recurrence and should receive extended-field EBRT. Although hypofractionated radiotherapy is not a standard of care, based on retrospective studies, it has demonstrated comparable safety with conventional fractionated radiotherapy when giving EBRT regimens of 45 Gy in 15 fractions [4] and 40 Gy in 16 fractions [28]. Several phase II prospective trials are currently underway to address this question.

**Figure 4.**
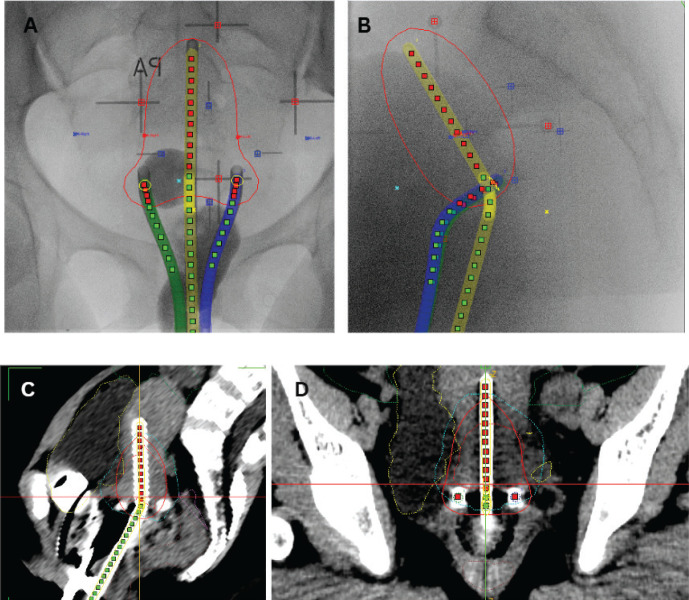
*Cervical cancer BT techniques*. (a)*: AP-PA view of* X*-ray image for 2D point A based BT planning.* (b)*: Sagittal view of* X*-ray image for 2D point A based BT planning.* (c)*: Sagittal view of CT-based 3D BT planning.* (d): AP-PA view of CT-based 3D BT planning.

BT is a key component in the curative treatment of LACC. Image-guided adaptive intracavitary/interstitial HDR BT is the standard approach, with applicators selected based on tumour extent and patients’ anatomy. BT should commence in the third to fourth week of EBRT as 1–2 sessions per week, prescribed to the ICRU Point A or high-risk clinical target volume for two-dimension (2D) or three-dimension (3D) BT techniques, respectively (Figure 4). Alternative fractionation schedules include 9 Gy in a single fraction weekly for 2 weeks, 6.5 Gy in a single fraction twice weekly for four fractions and 7 Gy in a single fraction twice weekly for three to four fractions [29].

Based on data from the INTERLACE Trial, induction chemotherapy (once-a-week intravenous (IV) carboplatin area under the AUC 2 and paclitaxel 80 mg/m² for 6 weeks) followed by standard cisplatin-based chemoradiotherapy is acceptable in fit patients with stage IIB cervical cancer with granulocyte colony stimulating factor support and ability to bridge to concurrent chemoradiation within 7 days of completing induction chemotherapy [13]. The use of adjuvant chemotherapy following definitive chemoradiation is not recommended [30].

### Metastatic and recurrent cervical cancer

**Figure 5.**
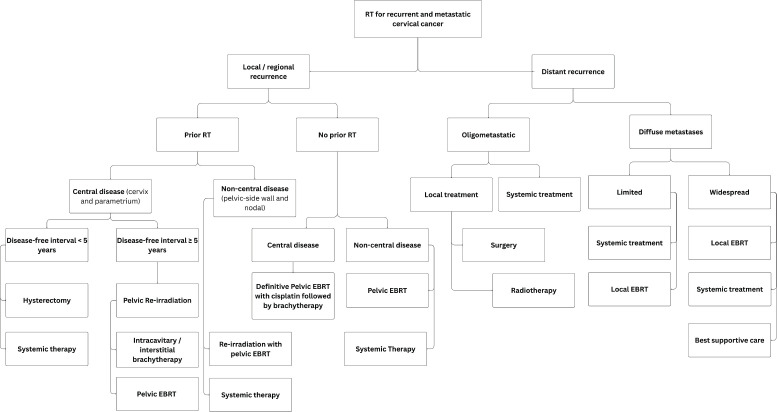
Use of RT for recurrent and metastatic cervical cancer.

For central pelvic recurrences after prior chemoradiation, salvage surgery—including pelvic exenteration or, less commonly, radical hysterectomy—can be considered, especially when the recurrent tumour is centrally located, ≤3 cm in size and in the absence of distant metastasis [31]. Palliative radiotherapy is administered to patients with metastatic or recurrent cervical cancer to alleviate symptoms that are not responsive to medical management. Common indications include pain, tumour haemorrhage, vaginal discharge and compression symptoms such as spinal cord compression, brain metastases or nodal and bone metastases. The prescribed total absorbed dose is typically 30 Gy, delivered in 10 fractions of 3 Gy per fraction, with five fractions per week over 2 weeks [32]. Alternative palliative radiotherapy regimens include 10 Gy in a single fraction to the whole pelvis, repeated monthly for 2–3 fractions [33]. Patients who attain a good partial response after the first two fractions of radiation may be considered for a single HDR intracavitary BT session, delivering 10 Gy to point A. Alternatively 20 Gy in 5 fractions to the pelvis [34]; and the 'QUAD Shot' regimen: 3.7 Gy twice daily (totalling 14.8 Gy per cycle) over 2 days, repeated monthly for up to 3 months [35] For localised central recurrences within the pelvis, re-irradiation with EBRT or BT is an option for selected patients depending on previous treatment, previous radiotherapy dose, time from prior radiotherapy and any persisting radiotherapy related toxicity (Figure 5).

### Radiotherapy procedures

#### 2D or conventional simulation

About 40%–50% of our cervical patients are planned with 2D conventional simulation procedure, which involves the use of bony anatomy and clinical judgement to define target volumes. The anterior – posterior (AP-PA) radiation field borders are superiorly: L4-L5 inter-disc space; inferiorly: lower border of the obturator foramen or 3 cm below the inferior extent of the vaginal disease; and laterally: 2 cm lateral to the true pelvis or pelvic brim to adequately cover the external iliac and obturator nodes. The lateral field borders maintain the same superior and inferior limits as for the AP-PA fields, with the anterior border at the anterior face (cortex) of the pubic symphysis and the posterior border at 1–1.5 cm beyond the sacrum hollow [36].

#### 3D or CT simulation

The bladder preparation protocol requires the patient to empty their bladder and then drink 500 mL (1 cup) of water 30 minutes before imaging and each treatment session, maintaining optimal hydration throughout treatment. The rectum preparation protocol involves following a high-fibre diet and taking laxatives, e.g., bisacodyl tablets 10 mg taken orally at bedtime for 3 days before computerised tomography simulation and throughout treatment. The patient will be positioned supine on the couch of the CT or conventional simulator using orthogonal lasers and immobilised with an individualised ankle and knee fixator, a head sponge and arms positioned over the chest. The RO will place a radio-opaque marker to localise the inferior extent of vaginal disease. The CT scan will be acquired in 3 mm slices from the T10 vertebral body to mid-femur, 35 seconds after administration of IV contrast (1 mL/kg of body weight).

#### Target volume delineation

Target volume delineation will be performed by a RO using the ICRU Reports 50, 62 and 83 [37, 38] and approved within 15 days after CT simulation. The Clinical Target Volume-1 (CTV1) will include the gross tumour volume (GTV), entire cervix and uterus. The CTV2 will include the upper vagina, to at least 2 cm below the lowest aspect of the GTV or cervix, and parametrium, while CTV3 will include obturator, internal iliac, external iliac and common iliac nodes [39–41]. Inguinal nodes should be included if the lower third of the vagina is involved, while para-aortic nodes will be contoured up to the level of the renal hilum in patients with involved para-aortic nodes or multiple pelvic LNs, including the common iliac nodal station. Nodes are contoured as 7 mm around visible vessels, excluding bone and muscle, following guidelines for nodal contouring [41]. For post-operative cases, a reduced target volume for EBRT resulting in a small pelvic field not including the common iliac nodes may be considered in low - and intermediate- risk patients with negative LNs on imaging and no LVSI. The Planning Target Volume (PTV) margins are 0.7 cm for the nodes, designated as PTV3, 1 cm for CTV2 – designated as PTV2 and 1.0–1.5 cm for CTV1 – designated as PTV1. The EBRT dose will be prescribed to the total PTV, which is the structure summing PTV1, PTV2 and PTV3.

#### Organs at risk (OAR) delineation

The OAR structures will include the bladder, rectum, femoral heads, bowel bag, small bowel (particularly for post-operative cases) and bone marrow. For extended-field radiotherapy, additional OARs include the spinal cord, kidneys and small bowel loops. These structures will be contoured using international atlases for OAR delineation [42] or AI-manual assisted delineation [43].

#### Radiotherapy plan evaluation

Plan configuration is performed by the planner (MP and/or Radiation therapist/dosimetrist) and approved by the RO. It involves several steps utilising the CT-CHOP criteria [44], reviewing the contours, targets, PTV coverage and conformity index, PTV dose heterogeneity and presence of hot spots, OAR constraints using the dose volume histogram (DVH) and clinical goal tool, and the prescription dose. The prescribed absorbed dose should be specified at the 100% isodose volume of the PTV or the ICRU reference point/isocentre. The planning goals will ensure that at least 98% of the PTV is covered by 95% of the prescription dose, without exceeding tolerances for OARs (Table 3).

#### Dosimetric considerations

Dosimetric calculations shall be performed by the MP on CT images or water phantom for 2D radiotherapy technique, with the absorbed dose within the patient/phantom geometry calculated using either the AAA or Acuros algorithm, with a maximum grid calculation resolution of 2.5 mm. An independent quality check of the radiotherapy plan will be conducted by an experienced MP to ensure accuracy and compliance with treatment guidelines.

#### Patient-specific quality assurance (PSQA) and independent dose verification

All IMRT and VMAT radiotherapy plans for each new patient shall undergo PSQA checks before treatment commences using portal dosimetry. The monitor unit and absorbed dose calculations shall be independently verified, using Mobius3D ensuring that the independently calculated dose is within 5% of the planned dose in terms of target coverage, DVH limits (Table 3), 3D gamma pass/fail rates and deliverability.

#### Concurrent chemotherapy

The RO will ensure optimal performance status (ECOG performance status of 0–2), bone marrow and renal function before prescribing concurrent chemotherapy. Cisplatin 40 mg/m² (capped at 70 mg) is ideally administered IV in 200 mL of normal saline (NS) is administered over 30 minutes, preferably 30 minutes to 2 hours before radiotherapy, once a week on a Monday or Tuesday, for at least 5–6 cycles during EBRT. Supportive pre-chemotherapy medications include Ondansetron 8 mg IV, Dexamethasone 8 mg IV and pre-hydration with 1,000 mL NS with MgSO₄ 2 g over 1 hour. Post-chemotherapy, the patient is hydrated with 1,000 mL NS over 1 hour with KCl 40 mEq and oral antiemetics such as Ondansetron 8 mg PO twice daily (BD) for 5 days or Granisetron 1–2 mg PO twice daily (BD) for 5 days.

#### Brachytherapy

Applicators should be inserted with the aid of an ultrasound scan, and fluoroscopy images taken to confirm applicator position, and generate a 2D BT plan. For small tumours or patients with no residual disease, template-based planning with a standard loading pattern can be considered. For stage IB3 and IIB, consider the use of 3D imaging with CT scan for 3D-based image-guided adaptive brachytherapy (IGABT) planning using the ICRU report 38 and 89 for the first implant and ultrasound guidance for subsequent implants (Figure 3). The goal of IGABT is to deliver a BT dose of 40 to 45 Gy EQD2 to reach a total EBRT+BT dose of 80–85 Gy for small cervical tumours and >85 Gy for large cervical tumours or tumours with poor response to EBRT, adenocarcinoma and stage III/IVA at initial presentation[7, 45–47]. Doses to OAR, especially the bladder, rectum, sigmoid and bowel should be tabulated using EQD2 Excel calculators and kept within tolerance (Table 4).

#### EBRT boost

An EBRT boost is recommended for patients who are ineligible for BT due to factors such as tumour size, poor performance status, vesicovaginal or rectovaginal fistula or vaginal stenosis. While there is no standard fractionation, we routinely administer an EBRT dose to PTV1 only, ranging from 14 to 20 Gy in 4 to 10 fractions. Recommended techniques include 3D conformal RT or IMRT; however, 2D radiotherapy can be considered in selected patients. Several studies are exploring the use of stereotactic body radiotherapy in the delivery of EBRT boost, due to its physical and radiobiological benefits; however, this remains inferior to conventional BT [48, 49].

#### Overall treatment time

The overall treatment duration should not exceed 56 days (8 weeks) [48]. Any unintended treatment interruptions must be compensated within the intended overall treatment timeframe using hypofractionation, as directed by the RO. EBRT and BT are scheduled concurrently, starting from Week 1 to 5, once a significant tumour reduction (tumour size ≤4 cm, without parametrial involvement) is achieved. EBRT and/or chemotherapy should be omitted on days of BT implants to avoid excessive toxicity.

#### Post-treatment follow-up

The RO shall conduct an end-of-treatment evaluation during the final week of radiotherapy. Post-treatment follow-up to assess quality of life and disease recurrence will occur at 4–6 weeks, then every 3–4 months for the first 2 years, followed by every 6 months up to 5 years, and then annually thereafter. In patients with uncertainty of clinical remission after 3 months post chemoradiation, a follow-up assessment in 2–3 months is recommended with consideration of biopsy to exclude residual disease. Clinical follow-up will be based on physical examination and symptom-guided imaging as appropriate and coordinated with the gynaecological oncology team. Routine laboratory tests and vaginal vault or cervical cytology tests are not recommended. Consideration of hysterectomy after definitive chemoradiation is not recommended, particularly when the patient has received IGABT.

#### Management of treatment-related morbidity

RT may result in acute toxicities such as gastrointestinal symptoms (e.g., diarrhoea and nausea), genitourinary irritation (e.g., frequency and urgency) and hematologic suppression during concurrent chemotherapy [50]. Long-term effects may include vaginal stenosis, fibrosis, chronic proctitis and radiation cystitis [50]. To mitigate these risks, patients should receive supportive care, counselling and be monitored closely during and after treatment. Vaginal dilator use and pelvic floor physiotherapy are encouraged post-treatment. Severe or persistent late effects should be referred to appropriate specialists.

## Discussion

This clinical practice guideline provides a comprehensive and pragmatic framework for the use of RT in the management of cervical cancer in Uganda. Developed in the context of resource-constrained settings, the guideline integrates contemporary evidence with practical adaptations that reflect the realities of radiotherapy delivery in Uganda and other LMICs. It emphasises evidence-informed decision-making, efficient resource utilisation and context-appropriate innovations to address Uganda’s significant burden of cervical cancer.

The recommendations align with core principles endorsed by leading professional bodies such as the American Society for Radiation Oncology, European Society for Therapeutic Radiation Oncology, American Brachytherapy Society and the National Comprehensive Cancer Network [51–53], namely prioritisation of tumour control, patient-centred care and toxicity mitigation. However, the guideline departs in its pragmatic approach to technology and infrastructure, such as the use of 3D simulation with the aid of pelvic MRI and PET/CT, and MRI-guided adaptive BT, as suggested remaining challenge for resource-constrained settings. Therefore, we adopted CT simulation for both EBRT and BT planning, which is cost-effective, consistent with guideline recommendations for resource-limited settings [7, 54].

One of the key strengths of this guideline is the incorporation of hypofractionated EBRT regimens, particularly in the management of LACC. These schedules reduce treatment duration, machine burden and patient costs—factors that are critically important in high-volume clinics where treatment delays compromise clinical outcomes. Similarly, the use of 2D or template-based BT planning, when 3D image-guidance is unavailable, demonstrates how high-impact treatment can still be delivered safely using simplified clinical workflows.

Despite these advances, implementation will require proactive engagement from institutional and national stakeholders. Frequent machine breakdowns, limited BT capacity, insufficient trained personnel and an unreliable power supply remain significant barriers. To mitigate these, deliberate investments in preventive maintenance, staff capacity building, stable power systems and procurement of essential planning and quality assurance tools must accompany clinical guideline rollout.

Importantly, the guideline serves as a foundation for integrating radiotherapy into Uganda’s broader cancer control plan. It is structured to inform policy development, resource allocation and workforce planning and guide the scaling up of regional radiotherapy services. Additionally, further research is needed to evaluate the clinical outcomes of these context-adapted strategies, particularly hypofractionation and CT-based BT, in Ugandan populations.

## Conclusion

This Uganda-specific clinical practice guideline offers a pragmatic yet evidence-based framework for improving radiotherapy access and quality in cervical cancer care. By leveraging hypofractionation, simplified BT and CT-based planning, it enables rational technology use without compromising outcomes. This guideline represents the prevailing standard of care regarding cervical cancer management in Uganda and manifests tremendous progress in techniques and potential outcomes, which is a result of continued partnerships, particularly with the International Gynaecological Radiation Oncology Consortium, which have provided continued technical guidance and mentorship.

## List of abbreviations

2D, 2-dimensional; 3D, 3-dimensional; CT, computed tomography; D2cc, is the minimal dose to the 2 cm^3^ (2 mL) of the organ at risk; EBRT, external beam radiation therapy; EQD2_10_, dose calculation to an equivalent dose of 2 Gy with an α-to-β ratio of 10; EQD2_3_, dose calculation to an equivalent dose of 2 Gy with an α-to-β ratio of 3; HDR, high-dose-rate; HR-CTV, high-risk clinical target volume; ICRU, International Commission of Radiation Units and Measurements; IMRT, Intensity modulated radiation therapy; LVSI, Lymphovascular space involvement; MRI, Magnetic resonance imaging; OARs, Organs at risk; PET, Positron emission tomography; RT, Radiation therapy; VMAT, Volumetric modulated arc therapy.

## Conflicts of interest

The authors declare that they have no conflicts of interest.

## Funding

There was no funding for this study.

## Figures and Tables

**Table 1.** Radiotherapy treatment options based on cervical cancer FIGO staging.

Category	FIGO 2018	Radiotherapy options	Comment
Early-stage cervical cancer	FIGO stages IA, IB1, IB2, and IIA1.	Intracavitary BT alone, with a dose of 60–65 Gy EQD2 prescribed to Point A, is recommended. For intermediate-risk patients, adjuvant EBRT with a dose of 45–50 Gy to the whole pelvis is advised. High-risk patients should receive adjuvant concurrent chemoradiation.	• In the postoperative management of early-stage disease, vaginal cuff BT is recommended for patients with close or positive margins, large or deeply invasive tumours, parametrial or vaginal involvement or extensive LVSI.
LACC	IB3–IIA2 and, IIB–IVA	Definitive external beam radiation with concurrent cisplatin (40 mg/m² weekly for 5–6 cycles), followed by intracavitary BT	• Induction chemotherapy should be considered in selected patients. However, adjuvant chemotherapy following chemoradiation is not recommended
Metastatic cervical cancer	IVB	Palliative radiotherapy (EBRT or BT) to pelvis and sites of metastases	• Ablative radiotherapy to oligometastatic sites, and definitive radiotherapy to the pelvis can be considered
Recurrent cervical cancer	Recurrence	Palliative radiotherapy for sites of recurrence	• Re-irradiation with EBRT or BT for central recurrences of the pelvis.

**Table 2.** 2018 FIGO staging for invasive cervical cancer [51].

Stage	Description
FIGO I	The carcinoma is strictly confined to the cervix
IA	Invasive carcinoma that can be diagnosed only by microscopy, with maximum depth of invasion ≤5 mm^a^
IA1	Measured stromal invasion ≤3 mm in depth
IA2	Measured stromal invasion >3 and ≤5 mm in depth
IB	Invasive carcinoma with measured deepest invasion >5 mm (greater than Stage IA); lesion limited to the cervix uteri with size measured by maximum tumour diameter^b^
IB1	Invasive carcinoma >5 mm depth of stromal invasion and ≤2 cm in greatest dimension
IB2	Invasive carcinoma >2 and ≤4 cm in greatest dimension
IB3	Invasive carcinoma >4 cm in greatest dimension
FIGO II	The carcinoma invades beyond the uterus, but has not extended onto the lower third of the vagina or to the pelvic wall
IIA	Involvement limited to the upper two-thirds of the vagina without parametrial involvement
IIA1	Invasive carcinoma ≤4 cm in greatest dimension
IIA2	Invasive carcinoma >4 cm in greatest dimension
IIB	With parametrial involvement but not up to the pelvic wall
FIGO III	The carcinoma involves the lower third of the vagina and/or extends to the pelvic wall and/or causes hydronephrosis or nonfunctioning kidney and/or involves pelvic and/or para-aortic LNs
IIIA	The carcinoma involves the lower third of the vagina, with no extension to the pelvic wall
IIIB	Extension to the pelvic wall and/or hydronephrosis or nonfunctioning kidney (unless known to be due to another cause)
IIIC	Involvement of pelvic and/or para-aortic LNs (including micrometastases)^c^, irrespective of tumour size and extent (with r and p notations)^d^
IIIC1	Pelvic LN metastasis only
IIIC2	Para-aortic LN metastasis
FIGO IV	The carcinoma has extended beyond the true pelvis or has involved (biopsy proven) the mucosa of the bladder or rectum. A bullous oedema, as such, does not permit a case to be allotted to Stage IV
IVA	Spread of the growth to adjacent pelvic organs
IVB	Spread to distant organs

a Imaging and pathology can be used, where available, to supplement clinical findings with respect to tumour size and extent, in all stages. Pathological findings supersede imaging and clinical findings

b The involvement of vascular/lymphatic spaces should not change the staging. The lateral extent of the lesion is no longer considered

c Isolated tumour cells do not change the stage but their presence should be recorded

d Adding notation of *r* (imaging) and *p* (pathology) to indicate the findings that are used to allocate the case to Stage IIIC. For example, if imaging pelvic LN metastasis, the stage allocation would be Stage IIIC1r; if confirmed by pathological findings, it would be Stage IIIC1p. The type of imaging modality or pathology technique used should always be documented. When in doubt, the lower staging should be assigned

**Table 3.** Planning aims for targets and OAR constraints for EBRT planning (adopted from the EMBRACE II protocol) [52].

Target	Hard constraint	Soft constraint
PTV-total (PTV50 or PTV45)	V95% >95% Dmax <107%	
PTV3	D98% >90% Dmax <107%	
CTV3	D98% >100%	D50% >102%
OAR	Hard constraint	Soft constraint
Bowel	Dmax <105% (47.3 Gy)	When no LN boost: • V40 Gy <100 cm^3^ • V30 Gy <350 cm^3^ When LN boost or paraaortic irradiation: • V40 Gy <250 cm^3^ • V30 Gy <500 cm^3^ • Dmax <57.5 Gy
Sigmoid	Dmax <105% (47.3 Gy)	Dmax <57.5 Gy
Bladder	Dmax <105% (47.3 Gy)	V40Gy <75% V30Gy <85% Dmax <57.5 Gy
Rectum	Dmax <105% (47.3 Gy)	V40Gy <85% V30Gy <95% Dmax <57.5 Gy
Spinal cord	Dmax <48 Gy	
Femoral heads	Dmax <50 Gy	
Kidney	Dmean <15 Gy	Dmean <10 Gy

**Table 4.** Planning aims for targets and OAR for BT [52].

Target	D90 CTV_HR_ EQD2_10_	D98 CTV_HR_ EQD2_10_	D98 GTVres EQD2_10_	D98 CTVI_R_ EQD2_10_	Point A EQD2_10_
Soft constraints	>90 Gy <95 Gy	>75 Gy	>95 Gy	>60 Gy	>65 Gy
Hard constraints	>85 Gy	-	>90 Gy	-	-

**OAR**	**Bladder D_2cm_^3^** **EQD2_3_**	**Rectum D_2cm_^3^** **EQD2_3_**	**Recto-vaginal point EQD2_3_**	**Sigmoid D_2cm_^3^** **EQD2_3_**	**Bowel D_2_cm^3^** **EQD2_3_**
Soft constraint	<80 Gy	<65 Gy	<65 Gy	<70 Gy	<70 Gy
Hard constraint	<90 Gy	<75 Gy	<75 Gy	<75 Gy	<75 Gy
